# Diffusible retinal secretions regulate the expression of tight junctions and other diverse functions of the retinal pigment epithelium

**Published:** 2008-12-08

**Authors:** Ru Sun, Shaomin Peng, Xiang Chen, Heping Zhang, Lawrence J. Rizzolo

**Affiliations:** 1 Department of Surgery, Yale University, New Haven, CT; 2 Department of Ophthalmology and Visual Science, Yale University, New Haven, CT; 3 Department of Pharmacology, Shandong University School of Medicine, Jinan, Shandong, P.R. China; 4 Department of Ophthalmology, 2nd Affiliated Hospital of Harbin University, Harbin, P.R. China; 5 Department of Epidemiology, Yale University, New Haven, CT

## Abstract

**Purpose:**

The retinal pigment epithelium (RPE) forms the outer blood-retinal barrier. It is unclear how culture conditions might alter barrier properties of isolated RPE. We examined whether retinal secretions that increase the barrier functions of tight junctions in vitro also make gene expression in general more in vivo-like.

**Methods:**

Chick RPE from embryonic day 7 (E7) and E14 were cultured on filters. Media conditioned by organ culture of E14 neural retinas was added to the apical medium chamber. RNA was isolated to probe the chick genome on Affymetrix microarrays, and expression was compared to native E14 RPE. Expression was further analyzed by quantitative real-time PCR immunoblotting and immunocytochemistry.

**Results:**

More than 86% of the genes expressed in vivo were expressed in basal culture conditions, including RPE-specific markers such as RPE65 and bestrophin. E14 retinal conditioned medium affected 15% of the transcriptome in E7 cultures (24% if serum was included), but only 1.9% in E14 cultures (12% with serum). Examination of 610 genes important for RPE function revealed that mRNAs for 17% were regulated by retinal conditioned medium alone in E7 cultures, compared to 6.2% for E14. For tight junctions, retinal conditioned medium had the most effect on members of the claudin family. Besides regulating mRNA levels, immunoblotting and immunocytochemistry suggested additional mechanisms whereby retinal secretions regulated protein expression and localization.

**Conclusions:**

Gene expression in primary cultures of embryonic RPE resembled the native tissue, but differentiation and the levels of gene expression became more in vivo-like when elements of the retinal environment were introduced into the medium bathing the apical side of the cultures. Albeit insufficient, retinal secretions promoted differentiation of immature RPE and helped maintain the properties of more mature RPE.

## Introduction

The retinal pigment epithelium (RPE) separates the outer layer of the neural retina from the capillaries of the choroid to form the outer blood-retinal barrier. Tissue interactions within the retina and choroid would be expected to regulate barrier properties along with other functions of the RPE. The RPE is the first cell type to differentiate in the retina, but as the neural retina and choroid develop around it, 40% of the RPE transcriptome will change its expression [1]. Many culture systems have been devised to study the RPE in isolation [2–9]. Each has strengths and weaknesses, but it is difficult to define what a differentiated cell should be. Rather than ask whether an RPE cell can become fully differentiated in isolation, it might be instructive to ask how an environmental interaction with the retina or choroid affects gene expression. Typically, an RPE-specific process or a few proteins or genes are used to determine whether a culture treatment improves or lessens the level of differentiation, or whether a culture model is suitable to test the physiologic response to a drug. However, different RPE functions need not be regulated in parallel, and signal transduction pathways function as an integrated web of many pathways. Although interventions that regulate cultured RPE are interesting in their own right, they occur in a context that does not exist in vivo. Barrier function is a measure of cell differentiation that reflects the interweaving of complex intracellular networks. We demonstrated that some aspects of barrier function can be enhanced by engineering the apical and basal environments to resemble the native environment [10]. Do our culture manipulations promote differentiation in general, or is it possible that as some aspects of cell behavior become more in vivo-like, others dedifferentiate?

We studied tissue interactions in a chick embryonic model for several reasons. Tissue is readily obtained from early and late developmental periods in quantities amenable to primary cell culture. Primary cell culture avoids the problem of dedifferentiation that results from adaptation to cell culture and passaging [11]. Retinal explants and conditioned medium regulate RPE functions [10,12,13]. The entire chick genome has been sequenced, which allows us to examine the entire transcriptome. A molecular definition for differentiation is provided by the published time course for RPE gene expression during normal development [1].

Among its various components, the blood retinal barrier requires tight junctions to retard transepithelial diffusion through the paracellular spaces. By freeze-fracture electron microscopy, tight junctions appear as a necklace of strands that encircle each cell. These strands reside in the apical end of the lateral membranes with the adherens junctions that bind each cell to its neighbors in the monolayer [14]. When the RPE is established in chick embryos on embryonic day 3 (E3), there are gap and adherens junctions in the apical junctional complex, but no tight junctions [15,16]. Even on E7, there are very few tight junctional strands [10]. The end of this stage is marked by an event in retinal development, the protrusion of photoreceptor inner segments through the outer limiting membrane [8]. During the intermediate phase of development (E9-E15) these strands grow in length and number to form an anastomosing network that completely encircles each cell. When this anastomosing network is complete, the junctions become functional [10,17], but the structure and the composition of the tight junctions continue to change through the late phase of development [18]. The late phase begins on E16, when outer segments of the photoreceptors begin to appear and ends with hatching on E21. The functions of the gap, adherens, and tight junctions are intertwined, and like the tight junctions, all members of the apical junctional complex are continuously remodeled throughout development [8,15,19,20]. The complex is a telling marker of differentiation, because it lies at the nexus of cellular pathways that regulate cell size, shape, polarity, proliferation, and barrier function [21–25].

One assay that reflects the function of tight junctions is the transepithelial electrical resistance (TER). The TER of RPE cultured from the early phase (E7) is low [26]. Even though the formation of tight junctions is induced by the artificial culture conditions, there are many discontinuities in the network of tight junctional strands [10]. Although E14 RPE has a continuous network of tight junctional strands in vivo, the strands become discontinuous when the cells are placed in culture. A serum-free medium that is conditioned by the organ culture of E7 neural retinas can increase the TER modestly, but E14 retinal conditioned medium is more effective [7]. This E14 retinal conditioned medium (rcSF3) seals the discontinuities of E7 and E14 cultured RPE, which results in a higher TER and more mature tight junctional structure [10]. Unless rcSF3 is added within days of culturing, RPE loses its ability to respond [7]. The effect of rcSF3 is quite different from the process of initial dedifferentiation followed by a months-long redifferentiation process that has been observed in cultured RPE [2–4,27]. Rather than an agent that induces cultured RPE to redifferentiate, rcSF3 appears to retard the initial dedifferentiation of primary cultures.

The current study addresses the hypothesis that E14 retinal conditioned medium maintains the differentiated state of E14 RPE that was placed in primary cell culture, and advances the differentiation of cultured E7 RPE. For the purposes of this study, we use the transcriptome of E14 RPE in vivo as a molecular definition of differentiation for this developmental stage. A variety of structures and pathways important for RPE functions are studied in some detail, but the major focus is on the genes and proteins of the tight junction.

## Methods

RPE was isolated from E7 and E14 White Leghorn chicken embryos (Sunrise Farms, Catskill, NY) and cultured on Transwell filters (Costar, Cambridge, MA), as described previously [26,28]. Briefly, the apical medium chamber contained SF3, a serum-free medium, or a retinal conditioned medium (rcSF3). The rcSF3 was prepared by incubating neural retinas that were isolated from E14 embryos in SF3 for 6 h at 37 °C. For all cultures, the basolateral medium chamber contained SF2 medium, which is SF3 that was supplemented with 45 µg/ml bovine pituitary extract (Upstate Biotechnologies, Lake Placid, NY). Medium was changed every other day, and cells were harvested on day 9, when the cultures were quiescent and had attained a stable TER [7]. TER was measured at 33 °C using endohm electrodes (World Precision Instruments, Sarasota, FL). Measurements were made in a modified SF3 in which the bicarbonate of DMEM was replaced with 20 mM HEPES (pH 7.2) on both sides of the culture (Invitrogen, Carlsbad, CA). Similar results were obtained in SF3, but the modified SF3 had the advantage that the pH was stable during the time the measurements were made.

### Hybridization to the microarray and quantification by the reverse-transcriptase polymerase chain reaction

Filters with cultured RPE were stored in RNAlatter (Qiagen, Valencia, CA) [10]. Total RNA isolation was performed with the RNeasy minikit (Qiagen), which was used according to the manufacturer's protocols. For hybridization to the microarray, three biologic repeats were prepared for each culture condition. For each biologic repeat, RPE was pooled from 6 filters bearing RPE that were plated on the same day. The quality of the total RNA was assessed by the Keck Center, Yale University (New Haven, CT) using formamide gels and a 2100 Bioanalyzer (Agilent Technologies, Santa Clara, CA). The Keck Center also performed the hybridization to microarrays of the chicken genome (Affymetrix, Santa Clara, CA) along with the initial statistical analysis for quality control.

Quantitative assays of specific mRNAs were performed using real time RT–PCR, as described [10]. Briefly, each of the biologic repeats used for quantitative RT–PCR was prepared separately from the biologic repeats used for hybridization to the microarray. The RNA concentration of each preparation was determined by fluorimetry using the Quant-iT Ribogreen RNA Assay Kit (Invitrogen, Carlsbad, CA) and a TBS-380 fluorimeter (Turner Biosystems, Sunnyvale, CA). The primer sets are listed in Table 1. Copy numbers were determined from a standard curve that was generated using linearized plasmid containing the relevant claudin or zonula occuldens-3 (ZO-3) sequence. Qualitatively, the data were reproducible, based on at least 3 independent experiments. Despite the use of 18S RNA as an internal standard, day to day variability made an absolute determination of copy number/ng total RNA very difficult. To minimize this variability and make comparisons between RNA samples from multiple culture conditions, all of the data within a single figure panel were collected on the same day, using the same master mix of reagents for the reverse transcriptase and polymerase chain reaction steps. Each data point is the average of 3–4 biologic replicates, and the standard error is reported.

**Table 1:** Primers used in this study for quantitative real-time RT–PCR.

**Name**	**Upstream 5′-3′**	**Downstream 5′-3′**
Claudin 1	GGATGGGTATCATCATCAGCA	AGCCACTCTGTTGCCATACC
Claudin 2	GAGCTCCTGTGCTGTCTCCT	ACTCACTCTTGGGCTTCTGC
Claudin 4L2	TGGATGAACTGCGTCTACGA	CATGATGATGGAGGTGACCA
Claudin 5	AGCCATTATTCCAGGTTCTCC	AAGGCAAGTGCATGTTACCG
Claudin 12	GCATGTAAGAGCCTGCCTTC	GTGTCACAACAGGGATGTCG
Claudin 19 (set 1)	GGTTTCTTTGGCATCATCGT	CTGTGTGGCGTACAAGGAGA
Claudin 19 (set 2)	TCTCCTTGTACGCCACACAG	CGGTAGTACTGCTGTCCTTGG
Claudin 20 (set 1)	TAACGCAGATGCAAGGACTG	GCAGACTCCTCCAGCAAAAC
Claudin 20 (set 2)	GACGGTCCCATTCAAGAGAA	ATGTCTCCAAAAACGCCAAA
ZO-3	GACACAAACATGGACGATGC	AATGCGTCCGGATGTAGAAG

### Data processing and statistical clustering

The data were first filtered to remove those probes not expressed at any time point. A probe is considered to be expressed at a time point if it is detected (i.e., labeled as “P” by GeneChip Operating Software; Affymetrix) in at least half of the replicates. For those probes expressed, the raw expression signal was log-transformed (natural logarithms). To identify significantly differentially expressed probes, we applied classic one-way ANOVA analysis.

The Short Time-series Expression Miner (STEM, version 1.2.2b) software [29] with default parameters was used for analyzing the set of regulated probes. Briefly, STEM implements a novel clustering method that depends on a set of distinct and representative short temporal expression profiles, and each probe in the data set is assigned to a profile with closest match. The expected number of probes assigned to each profile is estimated by permutation, and the statistically significantly overexpressed profiles are then identified. A probe is considered regulated by culture conditions if its p value is less than or equal to a threshold that yields a theoretical false discovery rate of 5% [30].

### Identification of protein pathways

For detailed analysis, we selected 13 cohorts of proteins that are important for RPE function. These included the visual cycle, melanogenesis, phagocytosis, lysosomes, tight junctions, adherens junctions, gap junctions, cytoskeletal elements and regulators, plasma membrane transporters, extracellular matrix proteins, extracellular matrix receptors, transcription factors, and signal transduction proteins [1]. To cast a broad net, we used Protein Lounge to identify candidates for these cohorts. Because of the limited annotation of the chick genome, some important candidates were omitted. Affymetrix, Ensembl, and DAVID software were used to correlate probe-set identifier numbers with the mRNA that they represent.

### Immunoblotting

RPE sheets isolated from E7, E10, E14, and E18 chick embryos or cultured RPE were solubilized on ice in 200 μl of 25 mM tris buffer, pH 8.0, containing 2% sodium dodecyl sulfate and 10 μl/ml Protease Inhibitor Cocktail (Sigma-Aldrich, St. Louis, MO). Melanin granules were removed by centrifugation. Detergent-resistant multimers of claudin were prevented from forming by adding 5 mM EDTA along with 50 μl of gel-loading buffer. The samples were incubated for 10 min at 37 °C and then for 5 min in a boiling water bath. Protein concentration was determined using the Micro BCA protein assay kit (Pierce, Rockford, IL). Equal amounts of protein were resolved by sodium dodecyl sulfate–PAGE and followed by immunoblotting. The level of α-tubulin staining was used as an internal standard to normalize each sample. Rabbit polyclonal antisera to claudins 1, 2, 4L2, 12, and 20 and to ZO-3 were raised by Antibody Solutions (Palo Alto, CA). Antisera to claudins 1, 2, and 12 were characterized previously [10]. Affinity purified antibodies were prepared by Antibody Solutions for claudins 4L2 and 20 and for ZO-3. Mouse monoclonal antibodies against α-tubulin were purchased from Zymed (San Francisco, CA). The immunoblots were developed using HRP conjugated secondary antibodies and ECL plus chemiluminescence reagent (Amersham Life Science, Arlington Heights, IL).

### Immunofluorescence

The subcellular distribution of claudins and ZO-3 was determined by indirect immunofluorescence. Cultures or tissues were fixed with 100% ethanol at 4 °C for 30 min, and labeled, as described previously [10,28]. ML-grade secondary antibodies conjugated with Cy2 and Cy3 dyes were purchased from Jackson ImmunoResearch Laboratories (West Grove, PA). Fluorescence images were acquired with an Axioskop (Carl Zeiss, Inc., Thornwood, NY), or a FluoView 300 confocal, microscope (Olympus, Melville, NY).

## Results

### Statistical clustering of the transcriptome based on the transepithelial electrical resistance

**Figure 1:**
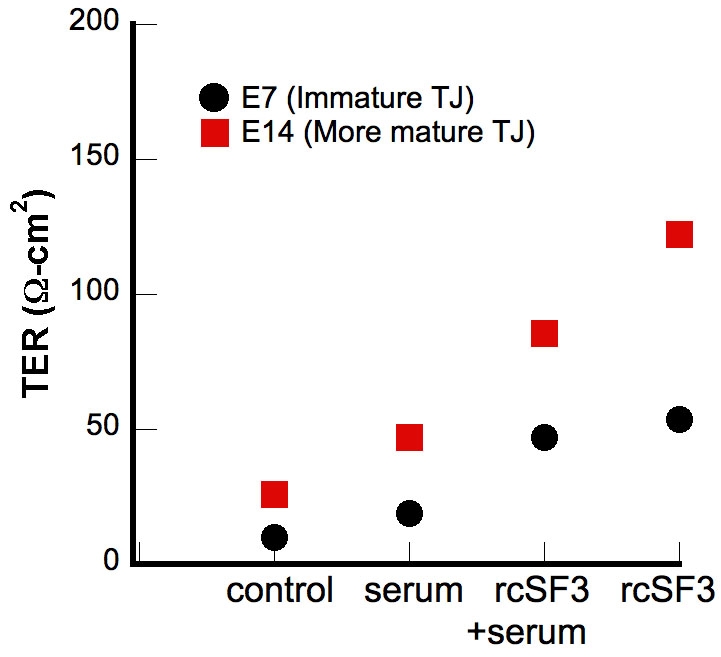
The transepithelial electrical resistance (TER) of cultured RPE is affected by serum and embryonic day 14 (E14) retinal conditioned medium (rcSF3). RPE was isolated from E7 or E14 cultures and cultured in control media (SF3, defined in the Methods, in the apical medium chamber and SF2, defined in the Methods, in the basal chamber) or with the following modifications: In some cultures, the basal media was supplemented with 2% FBS (indicated by “serum”). In some cultures, the apical medium replaced by retinal conditioned medium (indicated by rcSF3). Each data point is the average of 6–9 cultures. The standard error was smaller than the symbols.

The TER of the cultures used for the microarray analysis is reported in Figure 1. The data are arranged in the order of increasing TER. For each culture condition, the TER of E14 cultures was higher than that of E7. In each culture, E14 retinal conditioned medium (rcSF3) increased the TER 3–4 fold. These data confirmed earlier studies that demonstrated how the increase in TER correlated with a rearrangement of tight junctional strands into a functional network, and correlated with altered expression of the claudin family members that form the strands [10]. Serum by itself induced a modest increase in the TER, but slightly decreased the effect of rcSF3. Total RNA was isolated from these cultures and hybridized to microarrays of the chick genome. Three biologic repeats were analyzed for each culture condition. The raw data has been deposited in the GEO database under the accession number, GSE10538, and the mean for each probe set is included in Appendix 1.

**Figure 2:**
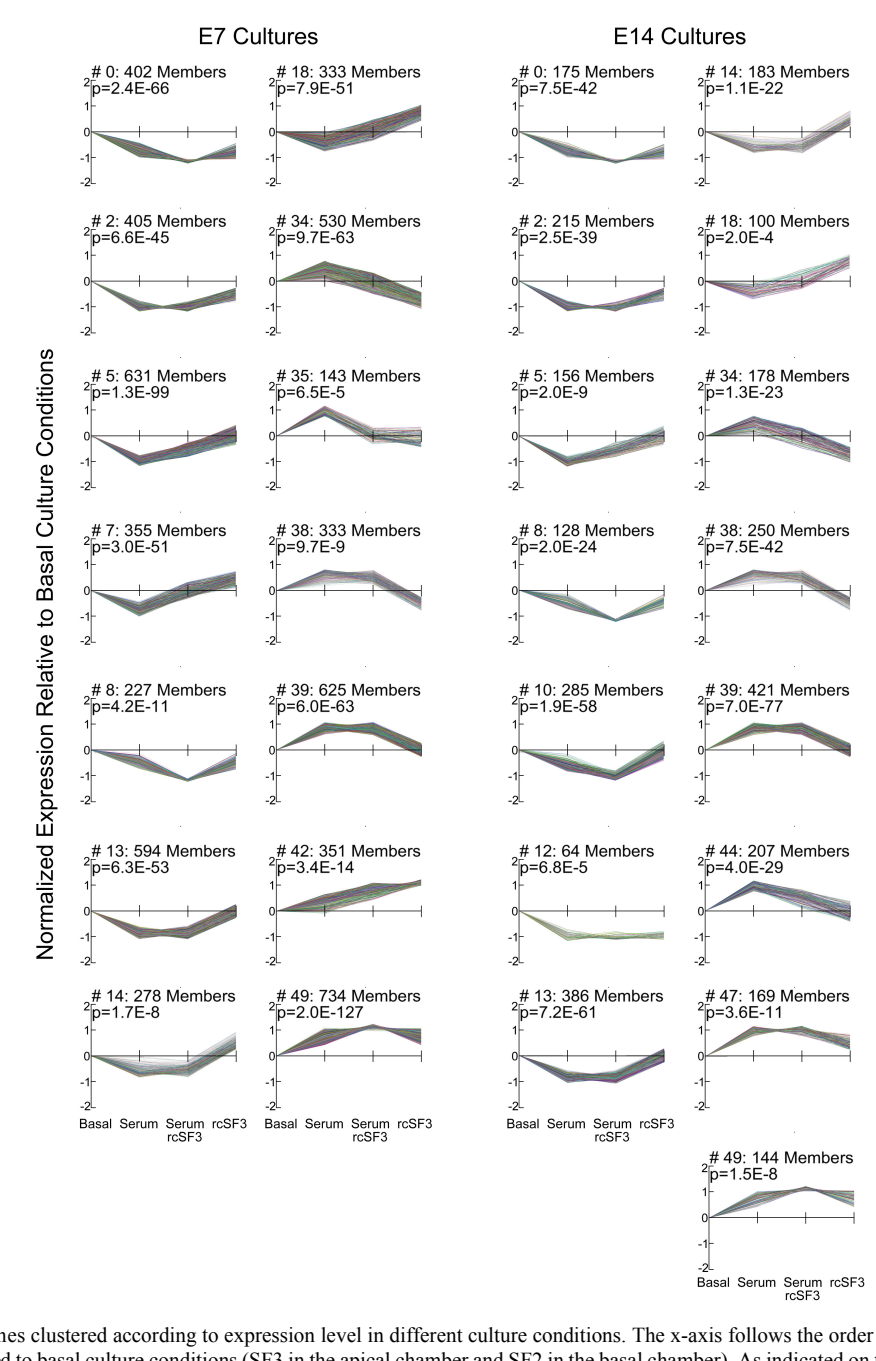
Genes clustered according to expression level in different culture conditions. The x-axis follows the order of Figure 1. Expression was compared to basal culture conditions (SF3 in the apical chamber and SF2 in the basal chamber). As indicated on the x-axis, cultures were supplemented with 2% fetal calf serum or rcSF3 replaced SF3. Data were transformed to allow high-expressing and low-expressing genes to be represented on the same graph. The p value from the statistical analysis and number of members for each cluster are indicated. Cluster memberships are listed in the Appendix 2. As summarized in Table 2, some gene clusters were affected by only serum or only rcSF3, but many clusters indicated interactions between these two stimuli.

To find genes that might be coordinately regulated, we used STEM software to cluster genes according to how their expression varied among the culture conditions indicated in Figure 1. A graphic representation of the statistically significant clusters is shown in Figure 2, along with the number of probe sets included in each cluster. The membership of each cluster is listed in Appendix 2. Some genes were regulated only by serum (clusters 13 and 39), but none were regulated only by rcSF3. As summarized in Table 2, the clusters indicated how apical (rcSF3) and basal (serum) stimuli could modulate each other’s effects on gene expression by augmenting or negating the effect of the other. Various classical markers of RPE differentiation were regulated in different ways that are summarized here and discussed in greater detail in the next section. In E7 cells, RPE65 mRNA (cluster 42) was present at low levels in basal culture conditions and increased with the progression of TER depicted in Figure 1. In E14 cells, RPE65 mRNA was expressed at higher levels that were unaffected by serum or rcSF3. By contrast, bestrophin mRNA was expressed at high levels regardless of culture condition. Despite the major contribution that members of the claudin family make to the electrical resistance of the tight junctions, they were distributed among clusters 13, 18, and 49 or were unaffected by culture conditions. In clusters 18 and 49, rcSF3 increased expression of the claudins, but the effects of serum and TER were not correlated. The tight junctional mRNAs and proteins will be discussed in greater detail below; the data thus far suggest that multiple mechanisms regulate the functions of tight junctions and that basal and apical stimuli collaborate to fine-tune function. When the interactions between serum and rcSF3 were pursued using quantitative real-time RT–PCR, a confounding factor was discovered. The same lot of serum was used for all the microarray data, but in the course of the RT–PCR experiments, different lots of serum were found to vary in their effects on TER and on gene expression. Accordingly, we pursued the apical limb of this regulatory pathway by examining E7 and E14 RPE that were maintained in SF3 or rcSF3. For these cultures, the results were reproducible using many preparations of RPE and rcSF3.

**Table 2:** Description of the statistically significant clusters represented in Figure 2.

**Description**	E7 Clusters*	E14 Clusters*
Affected only by serum	13, 39	13, 39
Affected only by rcSF3	None	None
Serum and rcSF3 oppose one another	7, 14, 18, 34, 35, 38	14, 18, 34, 38
Serum and rcSF3 reinforce one another	0, 8, 49	0, 8, 10, 49
Serum and rcSF3 modulate one another	2, 5, 42	2, 5, 10, 47
Serum and rcSF3 substitute for one another	None	12
Gene expression increases with increasing TER	42	None
Gene expression decreases with increasing TER	None	None

Cluster analysis showed that some genes were affected solely by serum, but none were affected by only rcSF3. For most genes there was an interaction between these two stimuli. Serum and rcSF3 could act synergistically to induce or repress gene expression. There were also examples where one opposed the action of the other. For a small subset of genes, the effects of serum and rcSF3 on expression paralleled the effects that they had on the TER. The asterisk represents the cluster numbers correspond to Figure 2.

### Genomic profile of the cultures

The microarray analysis indicated that the E7 cultures were more responsive to rcSF3 than the E14 cultures (Table 3). Each culture expressed most of the genes that are expressed during the course of normal RPE development between E7 and E18 [1]. A low percentage of the mRNAs detected in culture are not normally expressed in vivo. Serum and rcSF3 collaborated to regulate the expression of 24% of the transcriptome in the E7 cultures, but only 12% in the E14 cultures. A smaller percentage was regulated by rcSF3 in the absence of serum. Again more of these were regulated in the E7 cultures (Table 3). We focused on genes that are related to the epithelial functions of the RPE and its interaction with neighboring tissues. These genes for specialized function were selected as described in “Methods” and included genes for the visual cycle, melanogenesis, phagocytosis, lysosomes, tight junctions, adherens junctions, gap junctions, cytoskeletal elements and regulators, plasma membrane transporters, extracellular matrix proteins, and extracellular matrix receptors. We also considered the expression of transcription factors and regulators and signal transduction proteins that are expressed in native RPE. For E7 cultures, the proportion of these 610 genes that was regulated by E14 rcSF3 was similar to the total transcriptome (17% versus 15%). By contrast, for E14 cultures a higher percentage of this cohort was regulated compared to the entire transcriptome (6.2% versus 1.9%). To further distinguish the E7 and E14 cultures, we examined the 40 genes that were induced or repressed the most by rcSF3 (Appendix 3 and Appendix 4). Unexpectedly, three members of the ephrin (Eph) family of receptors were among the most affected genes. Together with their ephrin ligands, these tyrosine kinase receptors transmit bidirectional signals to regulate cell migration, adhesion, and tissue architecture [31,32]. Of this cohort of most regulated genes, 32% of the genes regulated in E14 were not regulated in E7 cultures. These include several transmembrane channels and transporters and extracellular matrix components that will be discussed in the next section.

**Table 3:** General characteristics of the E7 and E14 cultured RPE.

	**E7**	**E14**
Probe sets expressed	19,360	18,540
% of the probe sets detected in vivo (E7-E18)	89%	86%
% expressed in culture, but not in vivo	6%	5%
% affected by E14 rcSF3 when serum is present	24%	12%
% affected by E14 rcSF3 without serum	15%	1.9%
Genes important for RPE functions (340 examined)^1^		
Expressed within 2X in vivo level^2^	195	198
Not Expressed	34	39
Retinal condition medium affects expression^3^	40 (18)	13 (10)
Transcription factors and regulators (116 examined)		
Expressed within 2X in vivo level^2^	73	83
Not Expressed	1	8
Retinal condition medium affects expression^3^	8 (7)	5 (0)
Signal transduction genes (154 Examined)		
Expressed within 2X in vivo level^2^	70	83
Not Expressed	19	27
Retinal condition medium affects expression^3^	15 (13)	3 (7)

Total RNA from the indicated cultures was hybridized to microarrays of the chick genome. Data are presented for cultures maintained in basal media, and where indicated, for the effects of E14 retinal conditioned medium. ^1^Genes important for the epithelial-specific functions of the RPE, such as the visual cycle, phagocytosis, intracellular junctions, cytoskeleton, and transcellular transport were described in a study of RPE development [1]. ^2^Comparisons of the level of expression were made between cultures maintained in basal conditions and native RPE of the corresponding age. ^3^Because E14 retinal conditioned medium appears to promote differentiation in culture [10], levels of mRNA expression in E7, or E14, cultures supplemented with conditioned medium were compared to that of native E14 RPE. Indicated is the number of mRNAs for which the disparity in expression levels was lessened. Numbers in parentheses indicate mRNAs for which the disparity in expression increased.

The different effects of rcSF3 on the E7 and E14 cultures might reflect their different stages of development. The E14 retinal conditioned media might promote the differentiation of E7 RPE in culture, whereas its effects on E14 RPE might be to maintain the differentiated state that was already achieved at the time of isolation. Because a cell can only respond to a signal if it has the appropriate receptor, the differences might reflect a different complement of receptor, signal transduction, and feedback pathways. To explore whether E14 rcSF3 promoted the differentiation of E7 RPE, we compared expression in E7 cultures to the gene expression of native, E14 RPE.

**Figure 3:**
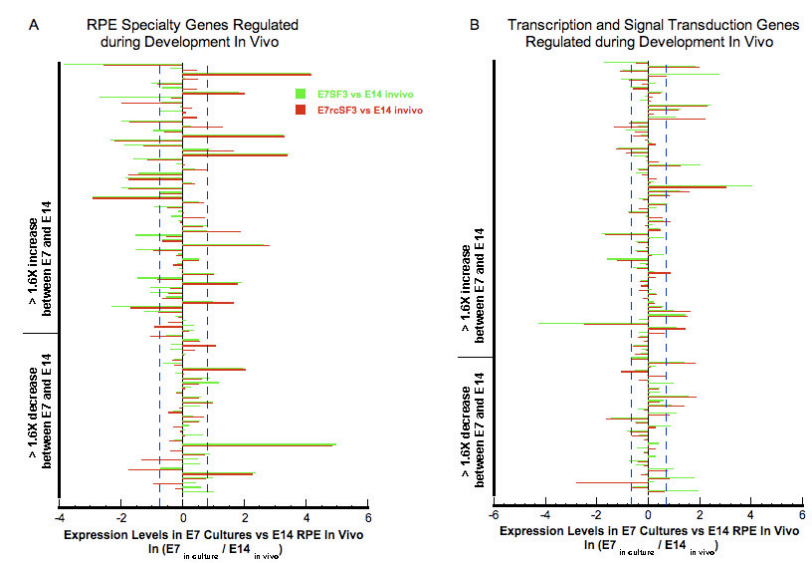
Effect of E14 rcSF3 on the maturation of E7 cultures. E14 rcSF3 made gene expression in E7 cultures more like that of E14 RPE in vivo. The following select genes were analyzed if their expression in vivo changed more than 1.6X during development [1]: genes involved in the visual cycle, phagocytosis, junctional complexes, cytoskeleton, matrix, and matrix receptors or plasma membrane transport (**A**); genes involved in the regulation of transcription or signal transduction (**B**). Each pair of green and red bars represents a single gene. Green bars indicate expression when E7 RPE was maintained in basal conditions (SF3); red bars indicate expression in retinal conditioned medium (rcSF3). The bars indicate the natural logarithm of the ratio (expression in culture)/(expression in native E14 RPE), where 0.0 indicates no difference between the E7 culture and native E14 RPE. A natural logarithm of 0.7 (dashed blue lines) indicates a 2X deviation of the E7 cultures from E14 RPE expression in vivo. Positive values indicate overexpression of a gene in culture. For most genes, the green and red bars are the same length, indicating no effect of rcSF3 on expression. For most of the genes that were affected by rcSF3, the red bar is shorter than the green bar, which indicates that expression levels approached the levels observed in native E14 RPE.

We focused on the 610 genes important for RPE functions that were summarized in Table 3. This list was further restricted by considering only those genes that were regulated during normal development in vivo [1]. Many of these mRNAs were expressed within twofold of E14 levels even in the absence of rcSF3 (Figure 3). Most of the genes that were regulated by rcSF3 changed their level of expression toward expression levels in vivo (E7_in culture_/E14_in vivo_=1.0), but several did not (Appendix 5). Some genes that are downregulated during differentiation were underexpressed in culture, and rcSF3 further decreased expression. In only two cases, rcSF3 caused the overexpression of a gene that is normally downregulated (axin 2 and guanine exchange factor, ARHGEF16). Similarly, some genes that upregulated during differentiation were overexpressed in culture, and rcSF3 further increased expression. In only three cases, rcSF3 caused the underexpression of a gene that is normally upregulated (collagen alpha 1(III), histone deacetylase 9, and V-ATPase C2 subunit).

**Figure 4:**
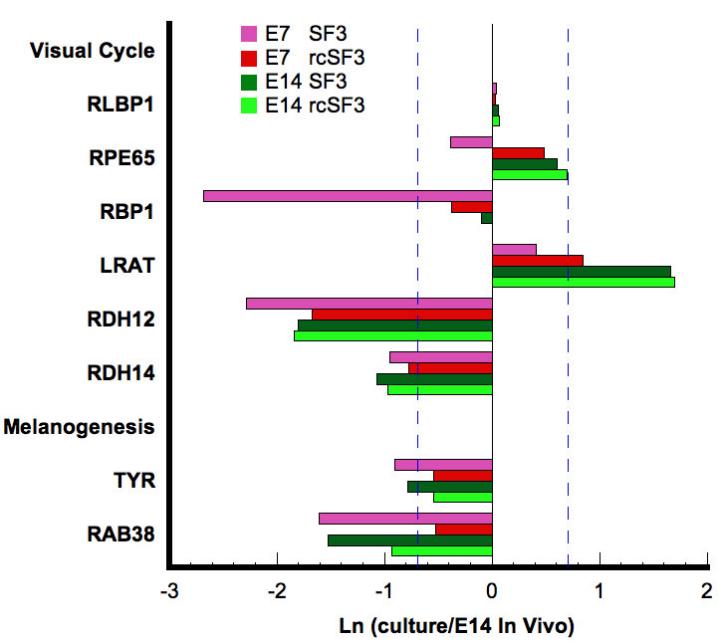
Effect of rcSF3 on the expression of genes involved in the visual cycle or melanogenesis. The ratio of (expression in culture)/(expression in native E14 RPE) is expressed as a natural logarithm. The dashed lines at ±0.7 represent a 2X deviation from expression on E14 in vivo. Complete gene descriptions and values for hybridization to the microarray are included in Appendix 6.

**Figure 5:**
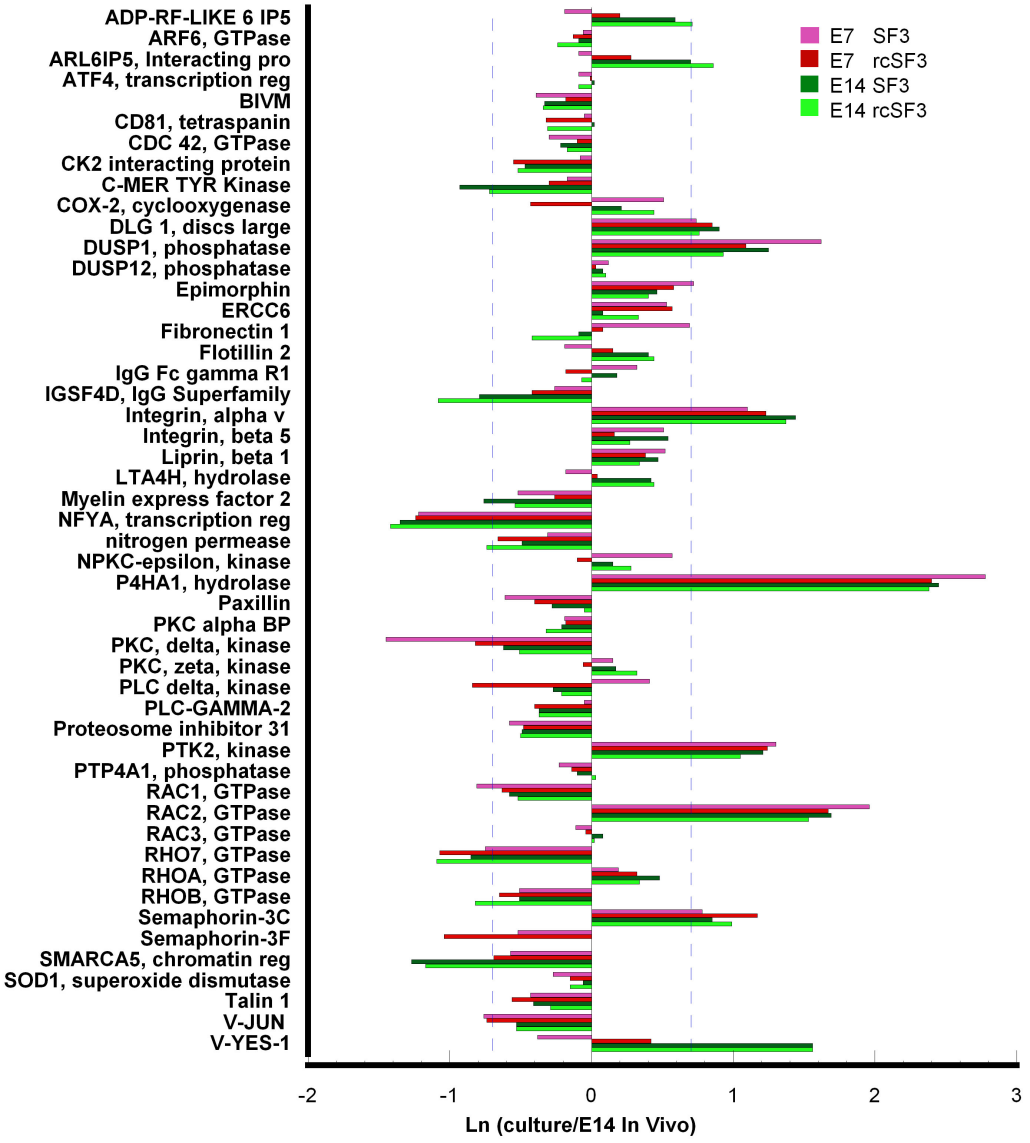
Embryonic age and rcSF3 had limited effects on the expression of genes involved in phagocytosis. The ratio of (expression in culture)/(expression in native E14 RPE) is expressed as a natural logarithm. The dashed lines at ±0.7 represent a 2X deviation from expression on E14 in vivo. Complete gene descriptions and values for hybridization to the microarray are included in Appendix 6.

To examine the effects of embryonic age and culture conditions in greater detail, we expanded the analysis, described in Figure 3 to include the E14 cultures. We examined the 340 genes, characterized in Table 3, as important for specialized functions of the RPE. Two themes emerged that were illustrated by the visual cycle and phagocytic related pathways. The first represents core structures or housekeeping proteins, such as lysosomes in the absence of a phagocytic challenge. Age of the embryos used to isolate the RPE and rcSF3 had minimal effects on the expression of lysosomal genes (data not shown). The second theme represents specialized functions of RPE such as phagocytosis, melanogenesis, and the visual cycle. A subset of these genes did change with embryonic age and were regulated by rcSF3, but rcSF3 affected more genes in the E7 cultures. The rcSF3 upregulated Rab38 in both cultures, but upregulated retinol binding protein 1 only in E7 cultures (Figure 4). Similarly, rcSF3 affected the expression of the phagocytosis-related genes, protein kinase C (PKC) delta and V-YES-1, but only in E7 cultures. On E14, most of the genes associated with the phagocytosis pathway were already expressed within 2X of expression in native E14 RPE (Figure 5). These themes are now examined with regard to genes important to the outer blood retinal barrier. The barrier includes the apical junctional complex and associated cytoskeleton, the plasma membrane pumps and channels that mediate transmembrane transport, and the extracellular matrix and their receptors. These proteins collaborate to establish the cellular polarity needed to establish transepithelial gradients and the paracellular seal that prevents those gradients from dissipating [23,24,33].

### Apical junctional complex

The tight junction that forms the paracellular seal between neighboring cells is a large assembly of transmembrane, adaptor, and cytoplasmic effector proteins [34]. The mRNAs for most of the relevant genes were expressed at levels within 2X of native E14 RPE. Of the exceptions, the expression levels of ZO-2, actin-related protein 3 (Arp3), and crumbs 2 (Crb2) were more in vivo-like in E14 cultures than in E7 cultures. Further, the expression of these mRNAs was regulated in E7 cultures by rcSF3. ZO-2 is an adaptor protein that links claudins and other transmembrane proteins to the effector proteins. Arp3 is part of a nucleation complex for actin filaments, which are concentrated at the apical junctional complex, but Arp3 also regulates the formation of actin filaments in other regions. Crb2 is part of a complex that regulates the polarized distribution of plasma membrane proteins. Most of the effects of age and rcSF3 were on the expression of the claudin family members.

**Figure 6:**
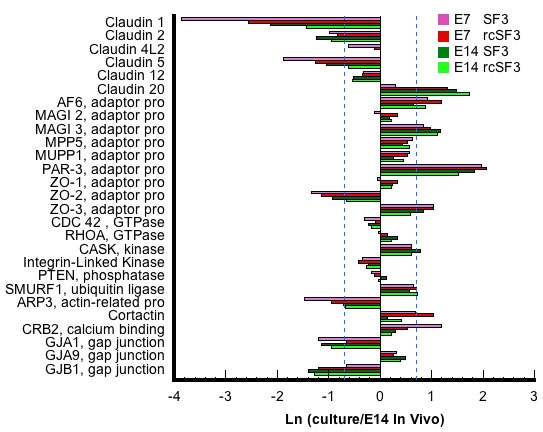
For tight junctions, rcSF3 and embryonic age primarily affect mRNAs of the claudin family. The ratio of (expression in culture)/(expression in native E14 RPE) is expressed as a natural logarithm. The dashed lines at ±0.7 represent a 2X deviation from expression on E14 in vivo. Complete gene descriptions and values for hybridization to the microarray are included in Appendix 6.

**Figure 7:**
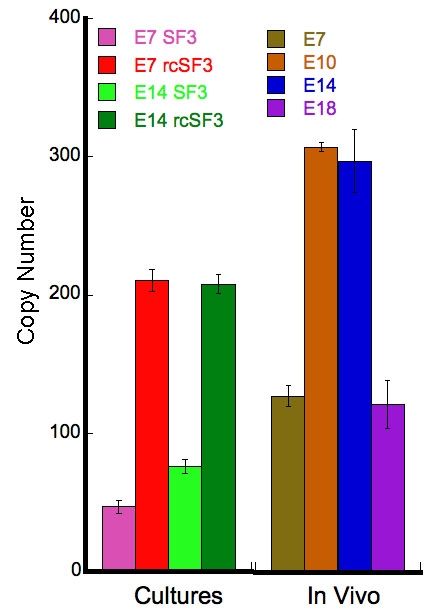
Expression of claudin 19 in vivo and in culture. Total RNA was extracted from native or cultured RPE. Native RPE was isolated from E7, E10, E14, and E18 embryos. For cultured RPE, RPE isolated from E7 or E14 embryos was cultured with SF2 in the basal medium chamber and either SF3 or rcSF3 in the apical medium chamber. In each reaction, 16.3 ng of total RNA was used. 18S RNA and GAPDH were used as internal controls (not shown). Quantitative RT–PCR was used to amplify claudin 19 mRNA using primer set 1 from Table 1. Similar results were obtained with primer set 2 (not shown). The level of expression on E18 was similar to the level found in E7 RPE, when few tight junctional strands are evident. Consistent with the peak of expression on E14, rcSF3 induced the expression of claudin 19 in E7 and E14 cultures. Error bars indicate the standard error of 3 independent experiments. The differences between SF3 and rcSF3 cultures were statistically significant (p<0.001). The differences between E7 or E18 RPE and E10 or E14 RPE were statistically significant (p<0.001).

Claudin family members form the strands observed by freeze-fracture electron microscopy and determine the ion selectivity of the tight junctions [35]. Of the 24 family members, 18 could be found in the chick genome and the same 7 were expressed by RPE in vivo and in vitro. The level of expression varied with age in vivo, and it varied among the cultures described here. With the exception of claudin 19, which is absent from the microarray, the mRNA for all of the claudin family members that are expressed in chick RPE are included in Figure 6. In general for genes affected by rcSF3, expression levels become more E14-like in the presence of rcSF3, but claudin 20 mRNA was overexpressed. In vivo, claudin 19 was transiently expressed with peak expression between E10 and E14 (Figure 7). Even at its peak, expression was very low compared to the other claudins. This result was unexpected because of the association of claudin 19 with ocular disease in humans [36]. The result was confirmed using two sets of PCR primers from different regions of the claudin 19 sequence. Consistent with the higher levels of expression on E14, the expression of claudin 19 mRNA increased in the presence of rcSF3 in E7 and E14 cultures.

**Figure 8:**
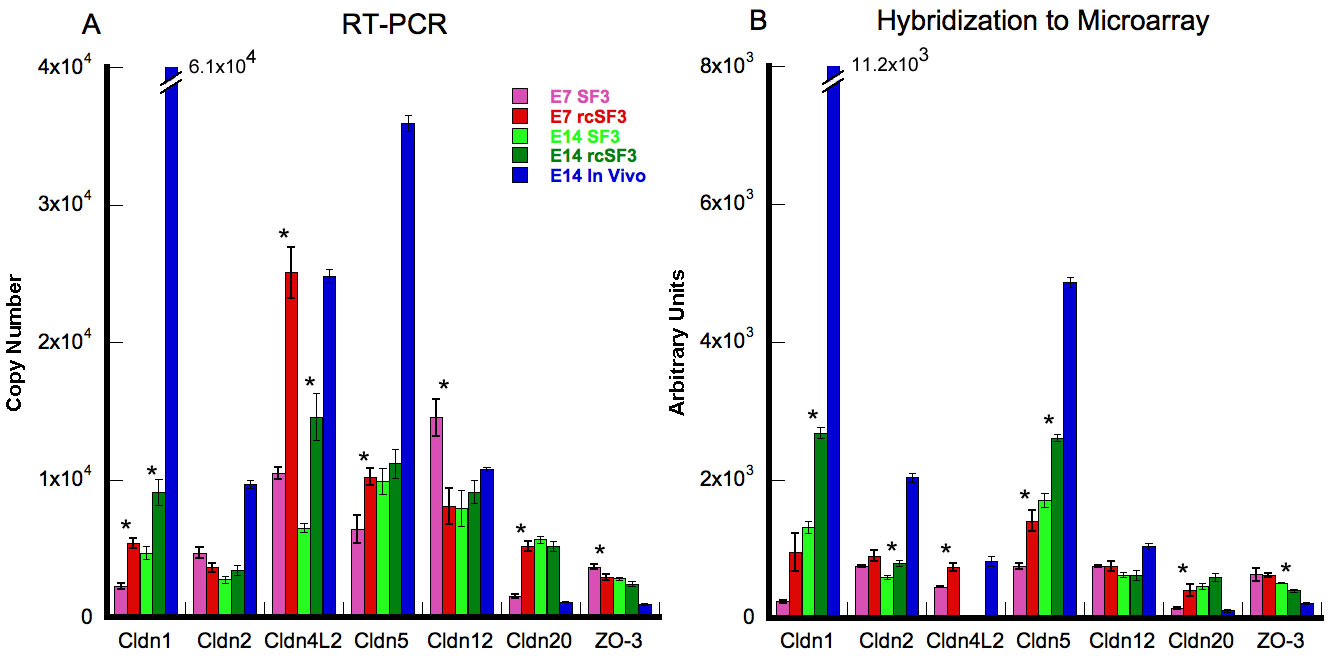
Comparison of quantitative RT–PCR and hybridization to the microarray for claudins and ZO-3. Total RNA was extracted from RPE that was isolated from E7 or E14 embryos and cultured with SF2 in the basal medium chamber and either SF3 or rcSF3 in the apical medium chamber. In each reaction, 16.3 μg of total RNA was used. 18S RNA and GAPDH were used as internal controls (not shown). For comparison, total RNA was also isolated from the RPE of E14 embryos. The effect of rcSF3 varied for the different claudins and was influenced by the age of the RPE at the time it was isolated from the embryo. Qualitatively, the data were similar to the data obtained by hybridization to the microarray. One notable exception was that claudin 4L2 was not detected by the microarray in the E14 samples, but was detected by RT–PCR. Error bars indicate the standard error of 3 independent experiments. Comparisons between SF3 and rcSF3 that were statistically significant (p<0.05) are indicated with an asterisk.

To determine whether the expression of the remaining claudins was accurately portrayed by the microarray, we used quantitative RT–PCR (Figure 8). To enable a comparison with the data in Figure 6, we included a sample of native E14 RNA. The trends revealed by the microarray were usually confirmed. Claudins 4L2 and 20 were overexpressed, but their level of expression on E14 is low in vivo before E18. The effects of rcSF3 depended upon the claudin. Like claudin 19, the mRNAs for claudins 1 and 4L2 increased in E7 and E14 cultures. The mRNAs for claudins 5 and 20 increased in E7 cultures, but the increase in E14 cultures for claudin 5 was not confirmed by RT–PCR. By contrast, claudin 2 mRNA was unaffected by rcSF3 on E7, but it did increase in E14 cultures. Although it was not predicted by the microarray, claudin 12 mRNA expression decreased in E7 cultures in response to rcSF3. Aside from the exceptions noted for claudins 5 (E14) and 12 (E7), and the absence of claudin 4L2 from the E14 microarrays, the microarray and RT–PCR data were in general agreement.

ZO-3 was included in this analysis because its expression sharply contrasts with ZO-1 and ZO-2 in vivo [1]. The latter mRNAs were expressed at constant levels during development, but the mRNA for ZO-3 was low in vivo until it increased on E18. In the current study of expression in culture, both the microarray and RT–PCR data demonstrated that ZO-3 mRNA was overexpressed in E7 cultures. There was little change in the expression of ZO-3 mRNA between E7 and E14 in vitro, or in response to rcSF3 (Figure 6 and Figure 8).

To examine whether mRNA expression for any of the claudins were coordinately regulated with one another or with putative regulatory proteins, we evaluated the statistical clusters described in Figure 2, Table 2, and Appendix 2. The genes listed in Figure 6 were stably expressed or distributed among a variety of clusters. The largest group that clustered together included Arp3, claudins 1, 4L2 and 5, and ZO-2 (E7, cluster 49), but they were not clustered on E14. In cluster 49, the combination of serum and rcSF3 led to the highest levels of expression for these claudins even though the TER in those culture conditions was lower than in rcSF3 alone. These claudins did not cluster together in the E14 cultures or during normal development in vivo [1] nor did they cluster with transcription factors, kinases, or phosphatases that might regulate their expression. However, the claudin and ZO mRNAs did cluster with members of the ubiquitin pathway that are believed to regulate the subcellular localization and half-life of specific membrane proteins [37–39].

### Steady-state levels and subcellular localization of junctional proteins

**Figure 9:**
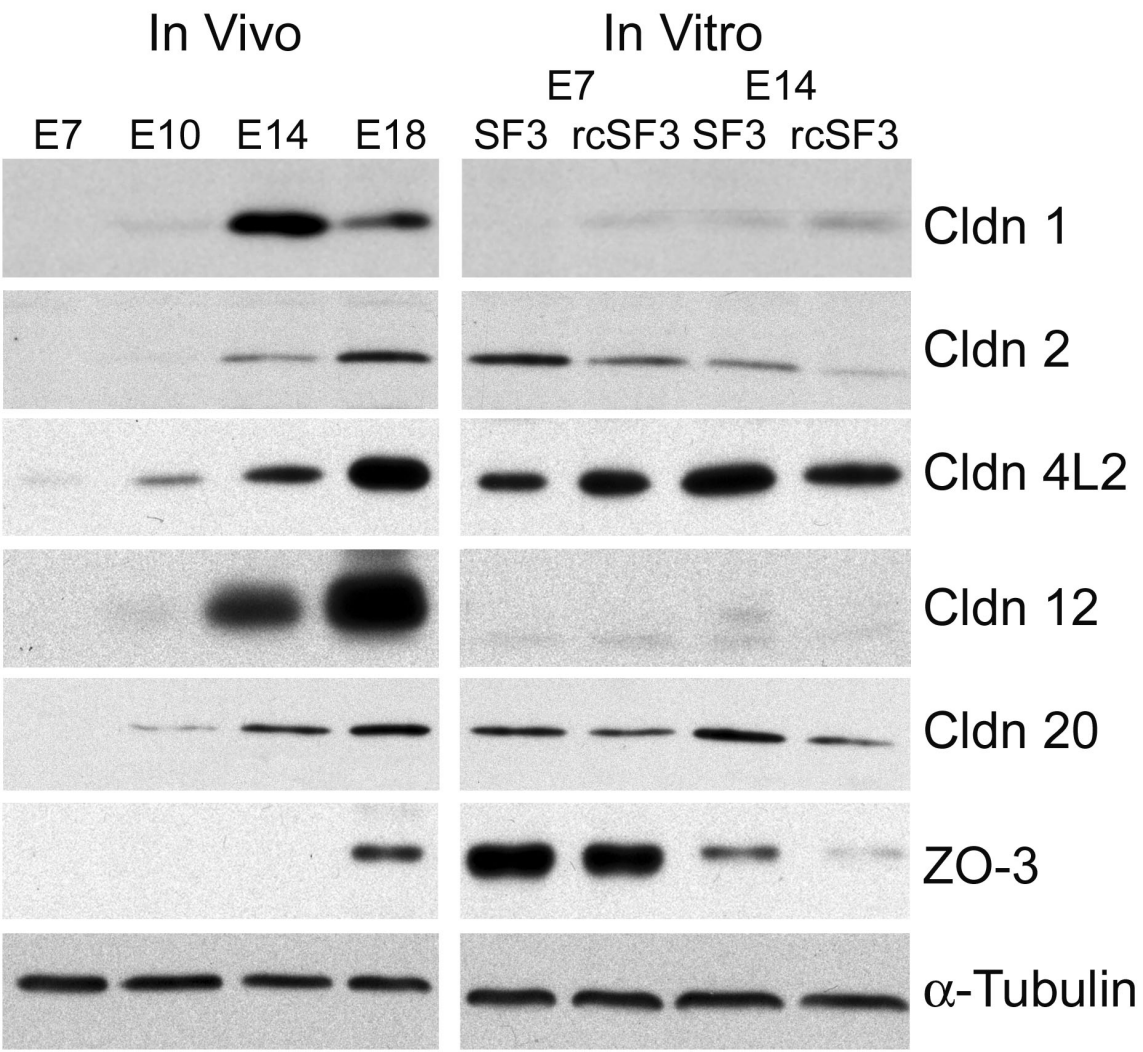
Protein was extracted from native or cultured RPE. Native RPE was isolated from E7, E10, E14, and E18 embryos. For cultured RPE, RPE isolated from E7 or E14 embryos was cultured with SF2 in the basal medium chamber and either SF3 or rcSF3 in the apical medium chamber. The proteins were resolved by electrophoresis, blotted onto membranes and probed with antibodies to the indicated protein. Antibodies directed against claudin 19 were not available. In preliminary experiments, proteins of the appropriate M_r_ were detected, and the corresponding peptide antigen could compete for binding of the antibody (data not shown). As inferred from mRNA levels, the relative amounts of different claudin family members varied among the different stages of development. The effects of rcSF3 on claudin expression were variable and, in some instances, was not predicted by changes in mRNA levels. The discrepancies between steady-state levels of mRNA and protein were especially large for ZO-3. The data are representative of 3 independent experiments.

Besides effects on gene expression, rcSF3 might regulate the half-life of the protein or its intracellular localization. To determine the effects of rcSF3 and embryonic age on steady-state protein levels, we further examined the claudins and ZO-3 by immunoblotting and immunofluorescence. For comparison, we examined native RPE during development and RPE in culture. For RPE in vivo (Figure 9), the time course for claudin and ZO-3 expression qualitatively followed the time course published for the corresponding mRNA [1].

In cultured RPE, the correlation between mRNA (Figure 8) and protein levels (Figure 9) suggested that additional mechanisms affected the steady-state protein levels. Protein extracts from freshly isolated RPE and cultured RPE were resolved on the same polyacrylamide gel and immunoblotted. The rcSF3 made the steady-state levels of some proteins closer to those of E14 in vivo. For some claudins (e.g., claudin 1), protein expression changed in parallel with mRNA levels. Previous studies demonstrated the same was true of claudin 5 [40]. In other cases, protein expression decreased despite an increase in mRNA (claudin 20 in E7 cultures and 4L2 in E14 cultures). For claudins 2, 12, and 20 in E14 cultures, protein levels decreased even though mRNA levels did not change. Even though there was very little difference in mRNA levels for ZO-3, ZO-3 was substantially overexpressed in the E7 cultures. Although E14 cultures expressed less, rcSF3 was effective in reducing ZO-3 expression further toward in vivo levels. It appeared that rcSF3 could exert control over both mRNA and protein levels to modulate the balanced expression of claudins and ZO-3.

**Figure 10:**
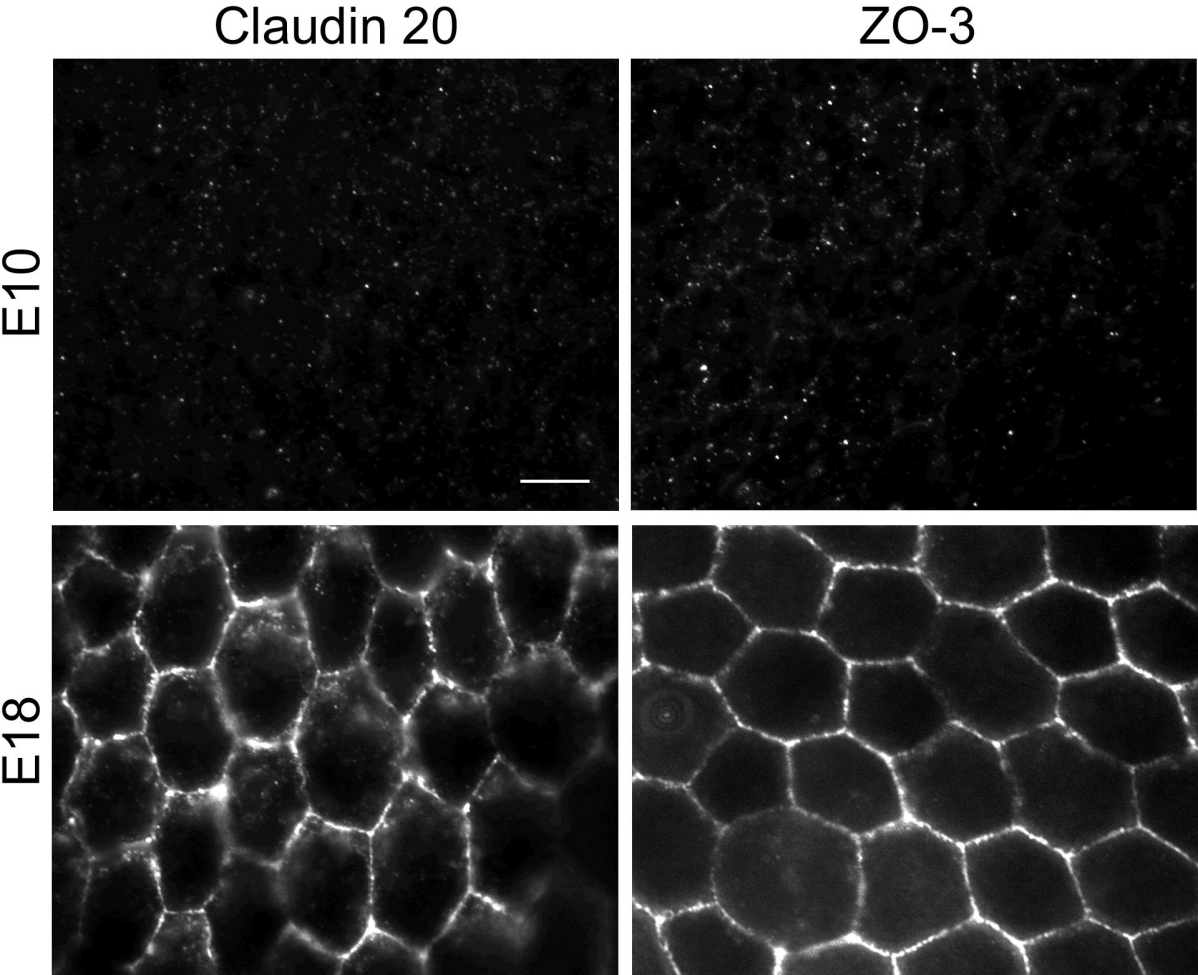
Expression and subcellular localization of claudin 20 and ZO-3 in vivo. Sheets of RPE and choroid were isolated from E10 and E18 eyes and fixed. The distribution of claudin 20 or ZO-3 was revealed by indirect immunofluorescence, as described in Methods. Neither protein could be detected in E10 RPE, but a clear signal was observed along the lateral membranes in E18 RPE. No signal was detected when the relevant peptide antigen was used to compete for binding of the antibody (data not shown). The scale bar represents 10 μm.

**Figure 11:**
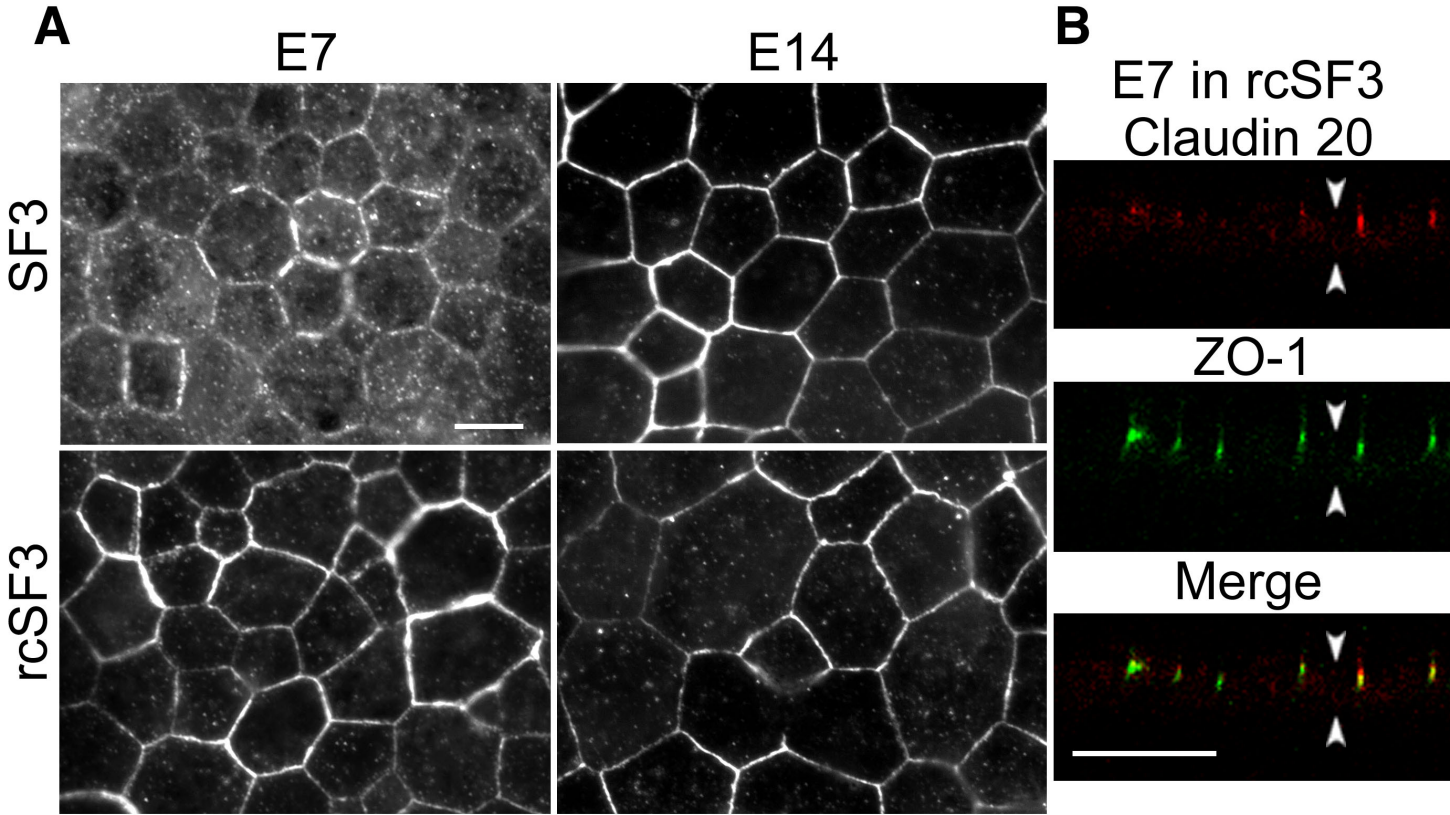
Expression and subcellular localization of claudin 20 in culture. RPE was isolated from E7 or E14 embryos and cultured with SF2 in the basal medium chamber and either SF3 or rcSF3 in the apical medium chamber. The cultures were labeled for claudin 20 (**A**) or double labeled for claudin 20 and ZO-1 (**B**). Under standard optics (**A**), claudin 20 was observed along the lateral membranes in each culture. When E7 RPE was cultured with SF3 in the apical medium chamber, a vesicular distribution was less evident when the RPE was cultured with rcSF3 in the apical medium chamber. Confocal microscopy (**B**) revealed the distribution of claudin 20 and ZO-1 in the XZ plane. Claudin 20 colocalized with ZO-1 in a junctional complex at the apical end of the lateral membranes, as indicated by the yellow in the merged image. In (**B**), arrowheads that point up indicate basal membrane, while arrowheads that point down mark apical membrane. The scale bar represents 10 μm.

**Figure 12:**
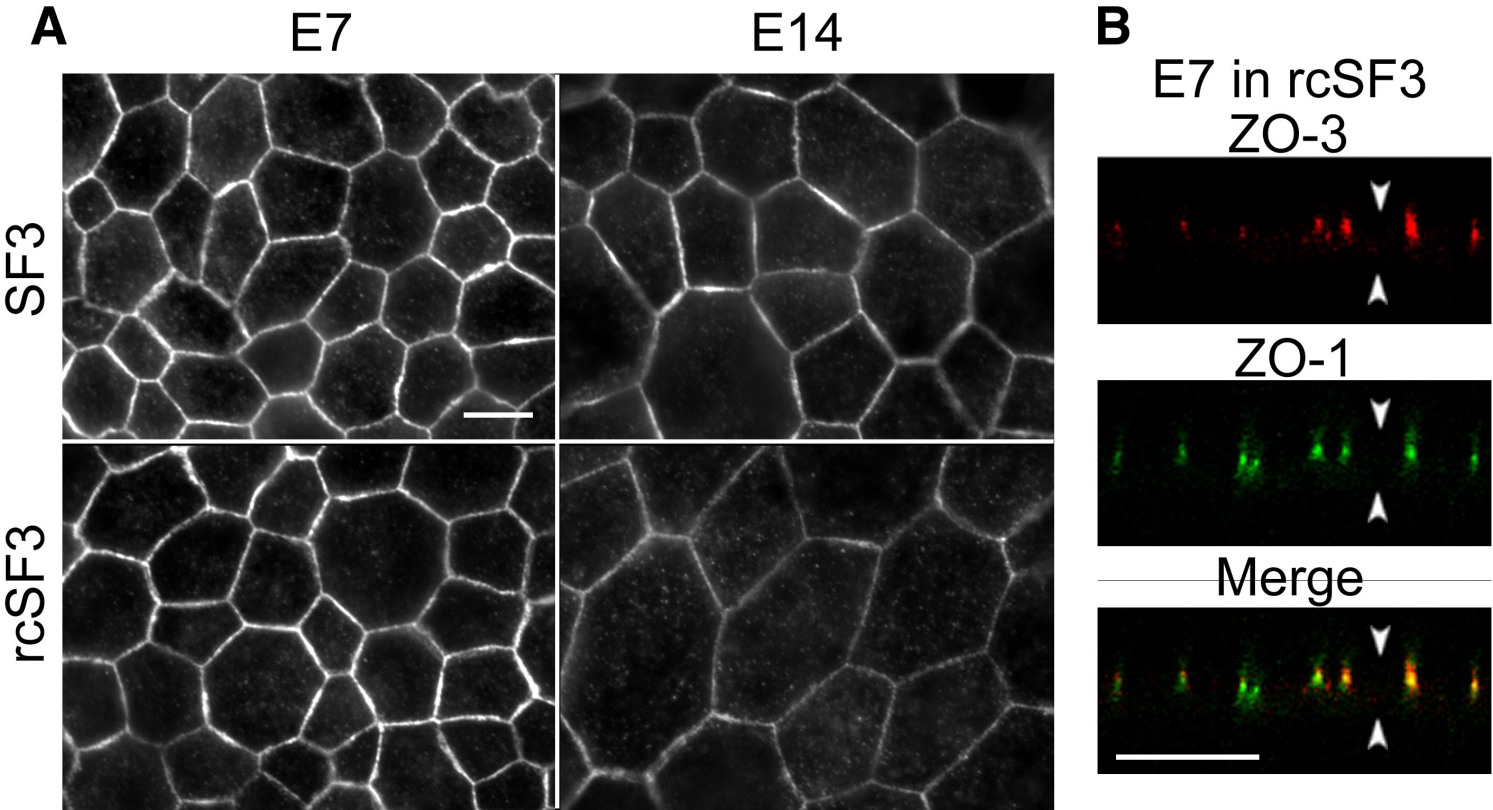
Expression and subcellular localization of ZO-3 in culture. RPE was isolated from E7 or E14 embryos and cultured with SF2 in the basal medium chamber and either SF3 or rcSF3 in the apical medium chamber. The cultures were labeled for ZO-3 (**A**) or double labeled for ZO-3 and ZO-1 (**B**). Under standard optics (**A**), ZO-3 was observed along the lateral membranes in each culture. There was no apparent effect by rcSF3 on the distribution of ZO-3. Confocal microscopy (**B**) revealed the distribution of ZO-3 and ZO-1 in the XZ plane. ZO-3 colocalized with ZO-1 in a junctional complex at the apical end of the lateral membranes. In (**B)**, arrowheads that point up indicate basal membrane, while arrowheads that point down mark apical membrane. The scale bar represents 10 μm.

Beyond effects on steady-state levels of mRNA and protein, rcSF3 might regulate the distribution of tight junction proteins between junctional and nonjunctional pools. Previous studies showed that claudins 1, 2, 5, and 12, and ZO-1, and ZO−2, localized to tight junctions in E7 and E14 cultures, but that there was a detectable intercellular pool of claudins 1 and 12 in the E7 cultures [10]. The current study provided data on the distribution of claudin 20 and ZO-3. We were unable to examine claudin 4L2, because the high background associated with this affinity purified antibody preparation led to ambiguous results (data not shown). In vivo, claudin 20 and ZO-3 were undetected on E10, but were clearly evident at RPE cell borders on E18 (Figure 10). In contrast to native tissue, claudin 20 was observed in E7 cultures both along lateral borders and in cytoplasmic vesicles (Figure 11A). This distribution appeared to shift to the lateral borders when the cells were maintained in rcSF3. In E14 cultures, claudin 20 localized to the tight junctions regardless of the presence or absence of rcSF3. When viewed in the XZ-plane by confocal microscopy, claudin 20 was concentrated in the apical junctional complexes that were identified by ZO-1 (Figure 11B). Together with the TER, these data indicate a distribution to the tight junctions. ZO-3 was found in the apical junctional complex in vivo and in vitro regardless of culture conditions (Figure 12).

### Other components of the apical junctional complex

**Figure 13:**
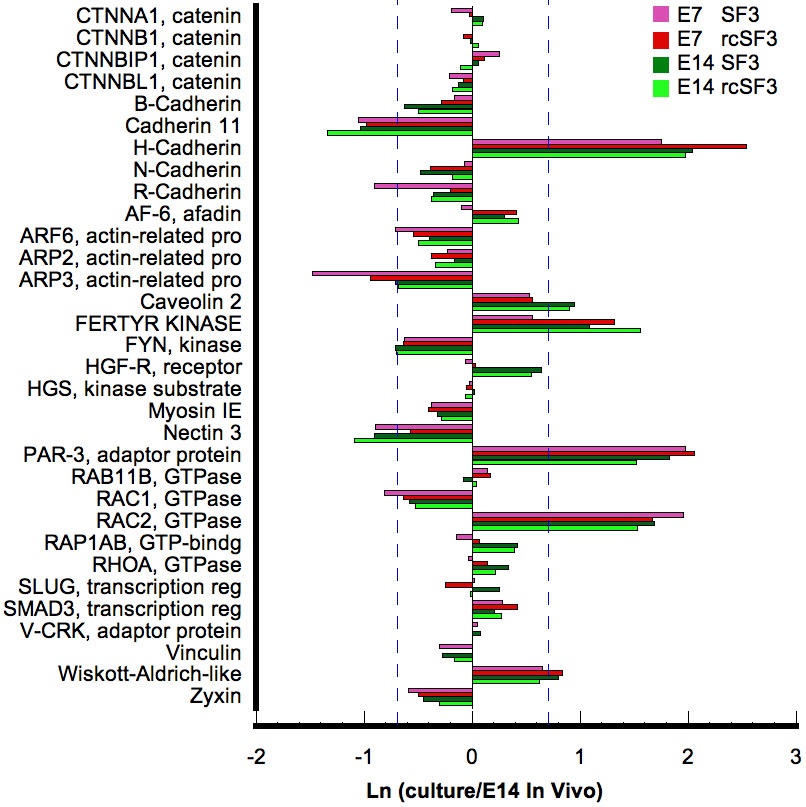
The effects of rcSF3 and embryonic age on the genes of the adherens junction are limited to Arp3, H-cadherin, and R-cadherin. The ratio of (expression in culture)/(expression in native E14 RPE) is expressed as a natural logarithm. The dashed lines at ±0.7 represent a 2X deviation from expression on E14 in vivo. Complete gene descriptions and values for hybridization to the microarray are included in Appendix 6.

To fully understand the function and regulation of the tight junctions, we have to consider its partner in the apical junctional complex, the adherens junctions, and the circumferential band of actin filaments that are linked to the complex. In culture, the mRNAs for most of the proteins known to affiliate with the adherens junction were expressed within 2× of the levels expressed in native E14 RPE (Figure 13). Most were not regulated by rcSF3, but notable exceptions were cadherin family members. Like the claudins, these are a family of transmembrane proteins that lend specialized functions to the junction, in this case by determining which external signals will be transmitted into the cell [41]. Like the claudins, cadherin family members are regulated during development [1,19,20]. Of the 5 cadherins expressed in culture, cadherin H mRNA was overexpressed and cadherin 11 was underexpressed. Cadherins H and R appeared to be regulated by rcSF3 in E7 cultures. These transmembrane proteins are linked to effector proteins via the catenins, whose mRNAs were expressed at in vivo levels and were not regulated by rcSF3.

The tight and adherens junctions are co-assembled by a complex process in which they share many proteins. Because rcSF3 removes discontinuities in the tight junctional strands, genes important for the assembly (Af-6, Jam A, Par-3, Par-6, and ZO-1) were examined previously in detail [42]. Those data demonstrated the mRNA expression was minimally regulated by rcSF3 in confirmation of the microarray data. The current study indicates that rcSF3 also fails to affect small GTPases that regulate assembly, including the repressor activator protein 1 (Rap1) and cell division cycle 42 (Cdc42; Figure 6 and Figure 13). As noted, Arp3, a regulator of actin dynamics was regulated by rcSF3 in both E7 and E14 cultures.

**Figure 14:**
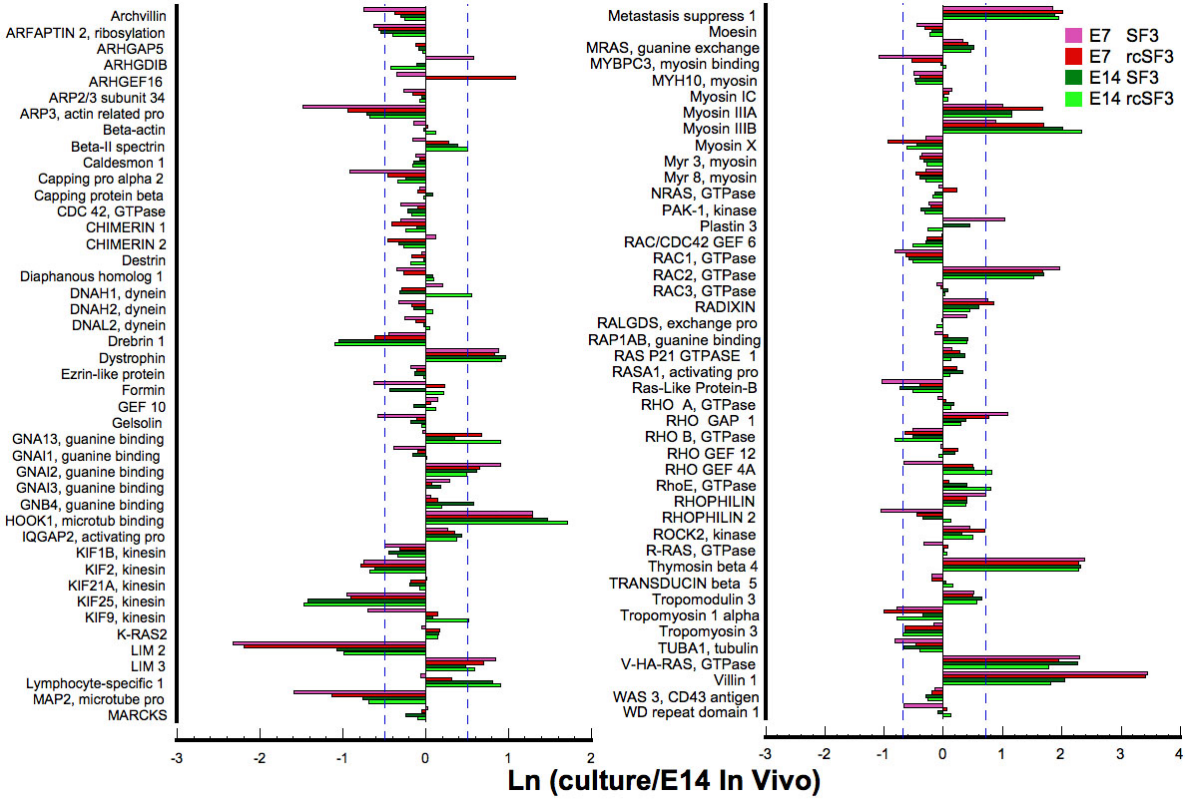
Effect of embryonic age and rcSF3 on genes that regulate actin and microtubule dynamics. The ratio of (expression in culture)/(expression in native E14 RPE) is expressed as a natural logarithm. The dashed lines at ±0.7 represent a 2X deviation from expression on E14 in vivo. Complete gene descriptions and values for hybridization to the microarray are included in Appendix 6.

The cortical ring of actin filaments, together with myosin II, can put tension on the apical junctional complex to regulate permeability [43]. Actin filaments and microtubles also contribute to barrier properties by regulating the movement of vesicular transport across the cell, vesicle-plasma membrane fusion events and the polarized distribution of membrane proteins [44–46]. The majority of the mRNAs for cytoskeletal proteins and their regulators were expressed within 2× of E14 levels, and few were affected by rcSF3 (Figure 14). Besides Arp3, the largest effects of rcSF3 were to increase expression in E7 of plastin3, WD repeat domain, Formin, RhoGEF4A, ArhGEF16, Map2, Kif3. Age-related differences included villin 1 (more overexpressed on E7) and Lim 2 (more underexpressed on E7).

### Plasma membrane

A second component of a blood-tissue barrier governs the transcellular pathway. Broadly speaking, this entails a polarized distribution of the receptors, pumps, channels and transporters of the plasma membrane. Matrix receptors help induce this polarity which enables vectorial transport of solutes across the cells.

**Figure 15:**
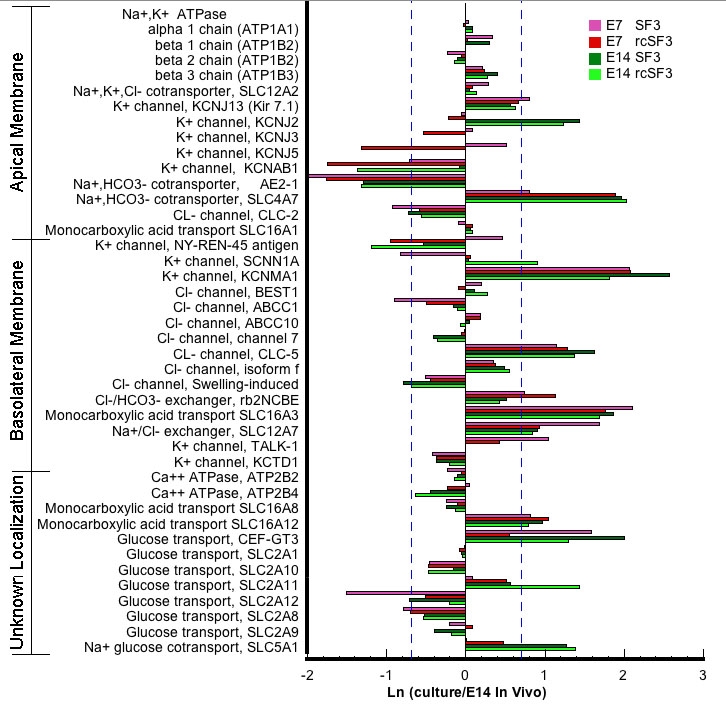
Effect of embryonic age and rcSF3 on genes that regulate transport across the plasma membrane. The ratio of (expression in culture)/(expression in native E14 RPE) is expressed as a natural logarithm. The dashed lines at ±0.7 represent a 2X deviation from expression on E14 in vivo. Complete gene descriptions and values for hybridization to the microarray are included in Appendix 6.

Unlike the aforedescribed cohorts of mRNAs, 36% of mRNAs for plasma membrane transport were not detected by microarray analysis of the cells in culture (Table 4). Because the energy source for transmembrane transport primarily comes from the ion gradients generated by the Na,K-ATPase, it is essential for cell survival and was expressed in all cultures close to the levels observed in vivo (Figure 15). By contrast the expression of the mRNAs for various ion transporters observed in vivo [1] were the least preserved (Table 4). Interestingly, the expression of the mRNA for several K^+^ conductance channels were downregulated by rcSF3. The expression of these channels was also downregulated during normal embryonic development [1]. The exception was KCNJ13, which is upregulated during normal development and stably expressed in culture.

**Table 4:** Ion transporters and channels expressed in vivo, but not detected in culture

**Apical membrane**	**Basal membrane**	**Unknown polarity**
Like Na^+^, K^+^, 2Cl^-^ cotransporter (splice isoform A)	Like Na^+^ conductance (voltage-gated, type 8)	ATP2B1, Ca^2+^-ATPase
*KCNJ3, K^+^-inwardly rectifying channel	L-type Ca^2+^ Channel	NDCBE1, Na^+^-driven Cl^−^HCO3 exchanger
*KCNJ5, K^+^-inwardly rectifying channel	CACNA1B, L-type Ca^2+^ Channel	KCND2, K^+^-voltage-gated channel
KCNA4, K^+^-voltage-gated channel	CACNA1C, L-type Ca^2+^ Channel	TASK2, volume-sensitive K^+^-channel
KCNC1, K^+^-voltage-gated channel	CACNB4, Ca^2+^ Channel	KCNC1, K^+^-voltage-gated channel
SLC24A2, Na^+^/K^+^/Ca^2+^ exchanger		*TALK-1, K^+^- channel
		SLC16A5, moncarboxylic transporter

For every cohort of genes described in this study, there were examples of genes that were expressed in vivo but not in culture. By far, ion transporters where the cohort that had the highest percentage of genes that cultured cells failed to express. Strikingly, most of these were potassium and calcium channels. The asterisk represents undetected in E14 cultures only.

An increase in transcellular glucose transport becomes essential for retinal function, as tight junctions develop to effectively block the paracellular pathway. In vivo, this entails the upregulation of a select group of facilitated glucose transporters and a sodium-glucose cotransporter. Previous studies demonstrated that GLUT 1 (SLC2A1) mRNA and protein, but not GLUT 3 (CEF-GT3), increased after E14, when the tight junctions became impermeable to glucose [47]. However, subsequent examination of the transcriptome during normal development revealed that the mRNA for many other glucose transporters were expressed. Although GLUT 3 and GLUT 12 (SLC2A12) are not developmentally regulated in vivo, the expression of their mRNAs was made more in vivo-like by rcSF3 in E7 and E14 cultures (Figure 15). SLC2A1, 8, 9, and 11 are upregulated during development in vivo, but only SLC2A11 was upregulated by rcSF3 in both E7 and E14 cultures. These are all facilitated glucose transporters, but one Na^+^ glucose cotransporter (SLC5A1) was expressed that could drive vectorial transport of glucose, and its expression increased during normal development. Notably, mRNA for the SLC5A1 was upregulated by rcSF3 and was greater in E14 than E7 cultures. These data support the hypothesis that this Na^+^ cotransporter drives the transport of glucose into the subretinal space after the blood-retinal barrier has formed.

The vectorial transport of lactate across the RPE monolayer is governed by a family of monocarboxylate transporters that have a polarized distribution in the plasma membranes [48]. The expression of these mRNAs (SLC16A1, 3, 8, and 12) was not affected by embryonic age or rcSF3 (Figure 15).

**Figure 16:**
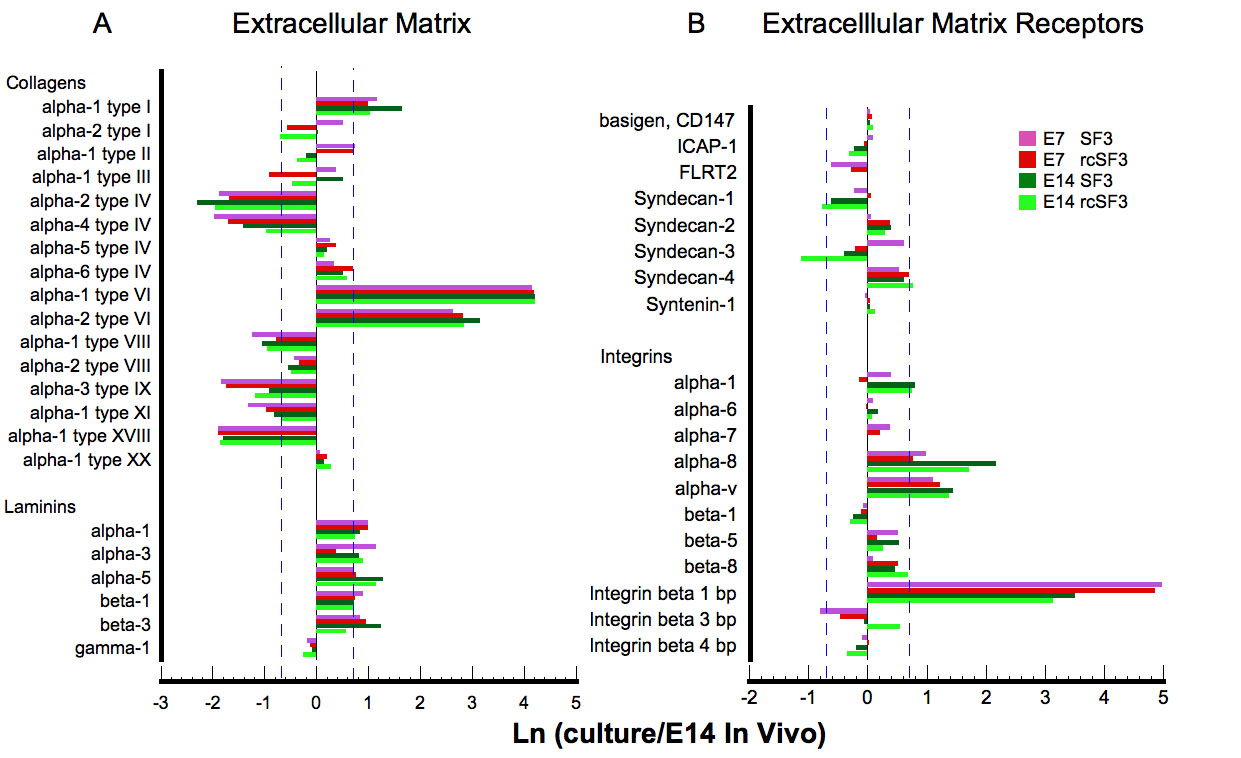
Effect of embryonic age and rcSF3 on genes for extracellular matrix components and their receptors. The ratio of (expression in culture)/(expression in native E14 RPE) is expressed as a natural logarithm. The dashed lines at ±0.7 represent a 2X deviation from expression on E14 in vivo. Complete gene descriptions and values for hybridization to the microarray are included in Appendix 6.

The expression of extracellular matrix receptors was minimally effected by rcSF3 (Figure 16). The largest deviations from normal expression were observed for an overexpression of the integrin β1 binding protein and integrin α_8_. Although there are no photoreceptor outer segments in the culture medium one of the receptors for them, integrin α_V_, was also overexpressed. However, its coreceptor, CD36, was not detected in E7 or E14 cultures. The extracellular matrix plays an important role in regulating cell behavior. Although many of the collagens are targeted for the inner layers of Bruch’s membrane, the collagen IVs and laminins would be in the RPE basal lamina, where they can influence RPE behavior. Notably, some of the collagen IV mRNAs were underexpressed, whereas many of the laminin mRNAs were slightly overexpressed.

## Discussion

The environment guides differentiation and maintains the phenotype of any cell. Pathology is often the normal response of a cell to an abnormal environment. Retinal detachment can cause multilayering of RPE [49], exposure of RPE to vitreous can cause proliferative vitreal retinopathy [50,51], accumulation of drusen in Bruch’s membrane and degeneration of the retina or choroid cause pathological responses in the RPE [52–54]. To retain native properties in culture, spontaneously or virally transformed RPE can mitigate this pathologic response [6,55]. Useful as transformed lines are, the most highly differentiated culture models derive from primary or secondary cultures of RPE that were isolated from developing eyes. To achieve this success, the natural environmental stimuli had to be replaced by highly specialized media [2–5,28]. But questions remain about how these cultures deviate from normal RPE and what signal transduction pathways mediate the effects of natural environmental stimuli. Our first step was to develop a molecular definition of differentiation [1].

Our hypothesis was that a natural stimulus would promote the differentiation of E7 RPE in culture and help maintain the phenotype of E14 RPE. Even in basal culture conditions, RPE expressed 85%–90% of the genes normally expressed, including high levels of RPE-specific proteins such as RPE65, bestrophin, and lecithin:retinol acyltransferase (LRAT). The cells had a cobblestone appearance and ZO-1 and occludin were localized to apical junctional complexes. These makers were incomplete measures of differentiation, as rcSF3 applied to the apical surface of the RPE promoted further differentiation of tight junctions [10]. The rcSF3 had an impact on many RPE functions by adjusting the expression levels of certain mRNAs to bring them into balance with other members of the same or interacting cellular pathways. Because each cellular pathway functions in the context of the others, the relative level of a gene’s expression is as important as whether or not the gene is expressed. As might be expected, the ability of rcSF3 to promote differentiation was incomplete. Previous studies demonstrated synergistic interactions between rcSF3 and stimuli in the basal environment [10] and the current study extends that observation to include serum (Figure 2, Table 2, Appendix 2, and Appendix 6). Statistical analysis demonstrated that the expression of many genes was modulated by the combined action of serum and rcSF3. Further, direct contact with the neural retina was needed to polarize the distribution of proteins such as integrin α_V_ and the Na,K-ATPase [12,13]. Among the genes most upregulated by rcSF3 were a family of receptors that mediate cell-cell interactions. The Eph receptors are inside-out and outside-in signal transducers that sense the environment to regulate the three-dimensional architecture of a tissue [31,32]. The expression of Eph receptors in RPE suggests they also mediate cell-cell interactions among the cells of the outer retina. Therefore, the analysis of rcSF3 is only the first step in understanding a web of interactions that regulate the RPE and integrate it into a functional retina.

E14 retinal conditioned medium had a greater impact on E7 than E14 cultures. For most of the affected genes, mRNA levels approached that of E14 RPE in vivo, but there were examples of the opposite effect. Serum was another source of stimuli that affected the expression of many genes. Cluster analysis demonstrated that serum and retinal conditioned medium often acted synergistically to modulate the effects of the other (Figure 2, Table 2, Appendix 2). This synergy merits further study, but was hampered by significant variability in lots of serum that became evident during studies by quantitative RT–PCR. By contrast, the effect of rcSF3 was consistent from preparation to preparation. Although an in-depth statistical analysis of the interactions of rcSF3 and this particular lot of serum might reveal genes that are coordinately regulated, we chose to focus on the effects of rcSF3 with the understanding that potential stimuli that might modulate rcSF3 were absent (e.g., serum, the secretions of choroidal cells and cell-cell contact with the neural retina).

The apical junctional complex is an important sensor of the external environment that regulates cell proliferation, polarity, and the transepithelial diffusion of solutes through the paracellular spaces. The rcSF3 regulates the assembly, fine-structure, and function of this complex [10]. Strikingly, the mRNAs of many components of the adherens, gap, and tight junctions were expressed within 2× of the levels normally seen in the in vivo tissue, and were unaffected by rcSF3. These included many of the adaptor proteins that link transmembrane proteins to effector proteins of the complex. The mRNAs that were regulated tended to be the transmembrane proteins that determine the functionality of the junctions. In the adherens junction, cadherins are receptors that transmit signals via the catenins. Two of the 5 cadherins detected in RPE were regulated by rcSF3, but the catenins were unaffected. In the tight junctions, claudins regulate diffusion across the paracellular pathway by determining the ion selectivity and electrical resistance of the junction [35]. Most of the claudin mRNAs were regulated by rcSF3 in a manner that depended on the claudin and the culture (E7 or E14). By contrast, the adaptor proteins that link claudins to the junctional network and many of the effector proteins were unaffected. With some minor deviations, quantitative RT–PCR largely confirmed the microarray data. Previous studies demonstrated that rcSF3 increased the TER of E7 and E14 cultures by closing discontinuities in the network of tight junctional strands [10]. A simple explanation would be that by increasing claudin mRNAs, there would be more of these strand-forming proteins to close the discontinuities. However, this simple explanation does not account for all of the facts. In chick RPE, the tight junctional network contains 4–5 parallel strands. Why did RPE in basal medium assemble a network with 4–5 parallel strands with discontinuities instead of a network with 2–3 parallel strands that was continuous? Further, rcSF3 actually lowered the protein level of some claudins at the same time it was sealing discontinuities (Figure 9). Therefore, the amount of claudins did not appear to be limiting. There was little effect of rcSF3 on the mRNA levels of putative assembly proteins, as was demonstrated by RT–PCR for the JAM family, AF-6 splice variants, Par 3 and Par 6 [42] or for various scaffold (or adaptor) proteins, as demonstrated in this study. Besides steady-state levels of mRNA, it appears that rcSF3 activated other mechanisms to regulate the distribution and steady-state levels of tight junctional proteins.

To better understand the effect of rcSF3 on the structure and composition of tight junctions, we examined the relationship of the steady-state levels of claudin mRNA and protein. During normal development, there appeared to be a parallel between the protein levels reported here and the mRNA levels reported earlier, although between E14 and E18 claudin 1 decreased, and claudin 12 increased, more that would be predicted from mRNA levels [1]. The culture data suggested that rcSF3 also regulated claudin translation or stability. For example, the mRNA levels of claudins 2, 12, and 20 were unaffected by rcSF3, but rcSF3 caused a decrease in protein level. For claudin 4L2 in E14 cultures, rcSF3 caused a decrease in protein level even though the mRNA increased. Therefore, rcSF3 was able to influence translation or protein degradation, either directly or indirectly. Earlier studies demonstrated that subcellular distribution was another potential point of regulation. There were large nonjunctional pools of claudins 1 and 12 in E7, but not E14, cultures maintained in rcSF3 [10]. Although this appeared to be an effect of embryonic age rather than rcSF3, rcSF3 did appear to regulate the subcellular distribution of claudin 20 in E7 cultures. The combined effect of these modes of regulation was that the steady-state levels of the various claudins relative to one another were different for E7 and E14 cultures. In particular, E14 cultures expressed more claudin 1 and less claudin 2. Because claudin 2 makes junctions leakier to Na^+^ [56], this might explain why E14 cultures exhibited a higher TER even though the fine-structure of the tight junctions is virtually the same for each culture [10]. These data support the model that tight junctions become functional during the intermediate phase of development, but that their selectivity continues to be modulated in response to the changing needs of the retina as it completes its differentiation [8]. The neural retina appears to regulate the expression of claudins through a variety of mechanisms.

Nontranscriptional regulation was particularly important for the zonula occludens family of adaptor proteins, ZO-1, ZO-2, and ZO-3. The mRNAs for ZO-1 and ZO-2 did not change during development even though the steady-state protein levels for both decreased dramatically [17,57]. By contrast, the mRNA for ZO-3 was undetectable in E7 RPE, but increased dramatically between E14 and E18, and ZO-3 protein followed suit. In both cultures, regardless of rcSF3, the mRNA was overexpressed, but ZO-3 itself was substantially overexpressed only in the E7 cultures. E14 cultures expressed much less and rcSF3 decreased steady-state levels of ZO-3 even more.

Activation or deactivation of small GTPases would be another means to regulate the assembly and structure of tight junctions. The assembly proteins are regulated by small GTPases, such as Rap1 and Cdc42, but rcSF3 had no apparent effect on their level of expression. Therefore, attention should focus on guanine exchange factors (GEFs) and GTPase activating proteins (GAPs) that might exert local control on these regulators. RhoGEF4A was upregulated 3× in E7 culture by rcSF3 and 11× in vivo. This GEF is important for neuronal morphogenesis during *Drosophila* embryogenesis [58]. RhoGEF4A activates Rho A, but not Rac 1 or Cdc42 to regulate filamentous actin. Several other genes that were affected by rcSF3-regulated actin dynamics. The importance of filamentous actin for RPE was illustrated by studies of the ARPE19 cell line. Culture media that improved barrier function also caused a redistribution of filamentous actin from stress fibers to circumferential bands affiliated with the apical junctional complex [59].

To relate the apical junctional complex to other pathways that mediate environmental interactions, we should consider the asymmetric nature of the RPE environment. Some of this asymmetry was reproduced in the culture model by growing the cells on a laminin-coated filter, with pituitary hormones supplied on the basal side and rcSF3 on the apical side. Aside from the absence of serum and the secretions of choroidal cells, the basal environment suffers by using commercial, laminin 1 to coat the filters. Laminin 1 is found in the extracellular matrix of embryonic tissues, but is replaced by other isoforms as epithelia differentiate [60,61]. The ability of cultured RPE to remodel the matrix was indicated by the laminins and collagen IVs that it expressed, as these are normal components of the basal lamina (Figure 16). The laminins were overexpressed in culture, which might reflect the cells attempt to remodel the matrix in an appropriate way, but the collagen IV isoforms expressed in vivo were underexpressed. Combined with the overexpression of β1 and α8 integrin chains, the basal signaling pathways of cultured RPE might deviate from normal cells. The absence of contacts with the neural retina might also affect the expression of transporters needed for vectorial transport, because the function of RPE is to regulate the environment for these interactions. Although rcSF3 affected the expression of several transporters, this cohort of genes showed the most substantial differences from gene expression in vivo. An exception to this observation was the expression of glucose transporters. The chick retina is more dependent on glycolysis than the mammalian retina. Previous studies demonstrated that the expression of glucose transporters correlated with the formation of tight junctions in vivo and in culture [47]. The microarray data extend this to a larger collection of facilitated transporters and a Na^+^ cotransporter that are regulated by rcSF3. It would be interesting to see if photoreceptor outer segments would induce the expression or regulated the distribution of the ion transporters.

The RPE of all species forms a blood-retinal barrier, but the specific properties of the barrier shows species variation [62]. This variability is reflected by species differences in the TER and transepithelial electrical potential. Whereas claudins 10 and 19 are prominent in human RPE [2,4,36], claudins 1 and 20 are prominent in chick. Nonetheless, the coordinated differentiation of the outer retina and choroid observed in chicks and mammals suggests there are fundamental, evolutionarily conserved mechanisms that regulate the assembly of the outer blood-retinal barrier [63–65]. This study demonstrates an apical stimulus, secretions of the neural retina, were more effective than a basal stimulus, serum, in regulating the functions of tight junctions and the expression of tight junctional proteins. Although rcSF3 was an effective agent to retard dedifferentiation of E14 cells in culture and promote the differentiation of E7 cells, rcSF3 alone was insufficient to direct RPE cell differentiation. The ability of serum to modulate the effects of rcSF3 suggests an important avenue for future studies of differentiation.

## Appendix 1. E7 and E14 Microarrays

Mean values for hybridization to the microarray is reported in arbitrary units as the natural logarithm. The p statistic for the one-way ANOVA analysis is indicated in column F. To access the data, click or select the words “Appendix 1.” This will initiate the download of an Excel archive that contains the file.

## Appendix 2. E7 and E14 Clusters

The top 50 from the STEM cluster program are listed. The basal condition is set at zero. The data are normalized to allow high expressing and low expressing genes to be represented on the same scale. The graph shows a schematic of how expression changes with culture conditions for each cluser. The x-axis is ordered according to columns C-F of row 7. The statistically significant clusters are colored. To access the data, click or select the words “Appendix 2.” This will initiate the download of an Excel archive that contains the file.

## Appendix 3. Genes most affected by retinal conditioned medium in E7 and E14 cultures: Upregulated genes

The 40 identifiable genes that were upregulated the most by E14 retinal conditioned medium are listed along with the level of expression relative to RPE maintained in basal conditions. Data represent the average of three microarrays that were probed with total RNA from independent experiments. Shading indicates genes that were upregulated in both E7 and E14 cultures. Italicized entries indicated genes that were regulated to the same or greater extent in E7 cultures as in E14 cultures, but were not among the top 40 regulated genes of E7. *Expression level in rcSF3 cultures relative to expression in SF3. ^†^genes that are included in the analyses of RPE specific pathways below. To access the data, click or select the words “Appendix 3.” This will initiate the download of a compressed (pdf) archive that contains the file.

## Appendix 4. Genes most affected by retinal conditioned medium in E7 and E14 cultures: Down-regulated genes.

The 40 identifiable genes that were down-regulated the most by E14 retinal conditioned medium are listed along with the level of expression relative to RPE maintained in basal conditions. Data represent the average of three microarrays that were probed with total RNA from independent experiments. Shading indicates genes that were down-regulated in both E7 and E14 cultures. Italicized entries indicated genes that were regulated to the same or greater extent in E7 cultures as in E14 cultures, but were not among the top 40 regulated genes of E7. *Expression level in rcSF3 cultures relative to expression in SF3. ^†^genes that are included in the analyses of RPE specific pathways below. To access the data, click or select the words “Appendix 4.” This will initiate the download of a compressed (pdf) archive that contains the file.

## Appendix 5. Quantification of the effect of rcSF3 on the differentiation of E7 RPE in culture.

Select genes characterized in Figure 3 and Table 3 were examined to identify those that E14 rcSF3 upregulated or down-regulated more than 2X in cultures of E7 RPE. For comparison, the second column indicates how gene expression would increase or decrease during normal development [1]. The third and forth columns indicate the level of mRNA expression in different culture conditions relative to expression in vivo on E14. The last, fifth, column indicates the level of expression in rcSF3 cultures relative to SF3 cultures. For many genes, the E14-derived rcSF3 increased or decreased expression in E7 cultures in parallel with an increase or decrease that occurred during normal development. For many genes, the culture/native E14 RPE was closer to the ideal of 1.0 in the rcSF3 cultures. To access the data, click or select the words “Appendix 5.” This will initiate the download of a compressed (pdf) archive that contains the file.

## Appendix 6. E7 and E14 Microarrays

Mean values for hybridization to the microarray is reported in arbitrary units as the natural logarithm. For comparison, column R reports expression in vivo on E14. The p statistic for the one-way ANOVA analysis is indicated in columns J and P. To access the data, click or select the words “Appendix 6.” This will initiate the download of an Excel archive that contains the file.
